# Vitrectomy for laser-induced full-thickness macular hole

**DOI:** 10.1186/s12886-021-01893-8

**Published:** 2021-03-14

**Authors:** Xin Wang, Ting Zhang, Rui Jiang, Gezhi Xu

**Affiliations:** 1 Eye Institute and Department of Ophthalmology Vision Sciences, Eye & ENT Hospital, Fudan University, 83 Fenyang Road, Shanghai, 200031 China; 2 NHC Key Laboratory of Myopia (Fudan University); Key Laboratory of Myopia, Chinese Academy of Medical Sciences, Shanghai, 200031 China; 3 Shanghai Key Laboratory of Visual Impairment and Restoration, Shanghai, 200031 China

**Keywords:** Laser injury, Macular hole, Vitrectomy, Optical coherence tomography, Optical coherence tomography angiography

## Abstract

**Background:**

To report the structure and visual outcomes of pars plana vitrectomy (PPV) for laser-induced full-thickness macular holes (MHs).

**Methods:**

This retrospective study enrolled 10 patients who underwent vitrectomy for MHs caused by laser injury. Best corrected visual acuity (BCVA), macular spectral-domain optical coherence tomography (OCT) and OCT angiography (OCTA) were used for assessment.

**Results:**

Four patients were injured by unexpected expose of an yttrium aluminum garnet (YAG) laser, and six patients were accidentally injured by a handheld laser. The MH minimum diameters (MDs) ranged from 55 to 966 μm (mean = 548.00 ± 286.10 μm), and BCVA ranged from 20/400 to 20/50 (mean = logMAR 0.87 ± 0.29) preoperatively. All 10 eyes underwent PPV, internal limiting membrane (ILM) peeling, and gas tamponade. All eyes demonstrated closure of the MH with different degrees of discontinuity of the outer layer of the retina, and four eyes exhibited serious retinal pigment epithelium (RPE) destruction. Postoperative BCVA values were significantly improved (mean = logMAR 0.55 ± 0.33; *P* = 0.032, t = 2.234). The mean BCVA of the destroyed RPE group was significantly worse than that of the non-destroyed RPE group both before and after surgery (*P* = 0.019; Wilcoxon signed rank test). Further, OCTA indicated choroidal ischemia in the laser-induced MHs.

**Conclusion:**

Vitrectomy can be successful in closing laser-induced full-thickness MHs and improving visual acuity. However, If RPE/choroid is involved in laser damage in addition to the outer retinal layer, this may indicate poor visual prognosis.

## Background

Reports of laser-induced macular holes (MHs), a special subtype of secondary MHs, have increased in recent years [1–3]. A careful review of medical history is essential for the correct clinical diagnosis. The type of laser can be neodymiumdoped yttrium aluminum garnet (Nd:YAG) or handheld lasers. Pars plana vitrectomy (PPV) has been used for the treatment of this condition in a few previous reports, but the number of such cases has remained small and the outcomes of this treatment have varied [4–7]. Herein, we collected 10 eyes of 10 patients who underwent vitrectomy for laser-induced MHs at the Eye and ENT Hospital of Fudan University to observe their structure and visual outcomes.

## Material and methods

This retrospective, observational study involved 10 patients treated by vitrectomy for full-thickness MHs caused by laser in the Eye Institute and Department of Ophthalmology at the Eye and ENT Hospital of Fudan University, China from 2013 to June 2018. This study was approved by the Medical Ethical Committee of the Eye and ENT Hospital of Fudan University. Each patient or his/her legal representative signed the informed consent to render treatment prior to surgery. The initial and follow-up evaluations included Snellen best corrected visual acuity (BCVA) measurement, slit-lamp biomicroscopy, spectral-domain optical coherence tomography (SD-OCT) (Spectralis OCT; Heidelberg Engineering, Heidelberg, Germany, or Cirrus; Carl Zeiss Meditec, Inc., Dublin, CA, USA), and PLEX Elite 9000 swept source OCT angiography (OCTA, Carl Zeiss Meditec, Inc., Dublin, CA, USA). The minimum linear diameter of the MH in horizonal OCT scans was defined as minimum diameter (MD). The base diameter (BD) was measured at the level of the retinal pigment epithelium crossing the center of the MH. Serious RPE destruction or destroyed RPE was defined the RPE layer under MH disappeared or couldn’t been recognized completely in SD-OCT.

All patients were treated by standard 23-gauge PPV combined with indocyanine green-assisted internal limiting membrane (ILM) peeling, and fluid-air exchange or inert gas (SF6 or C3F8) tamponade by multiple doctors. Among them, inverted ILM flap [8] technique was used in patient 1, 3, 4 and 9. The choice of tamponade was made depending on each doctor’s preference and the availability of tamponade. The patients remained prone for at least 5 days following surgery.

Data were analyzed using statistical software (SPSS version 18, IBM-SPSS, Chicago, IL, USA). Snellen BCVA value was converted to the logarithm of the minimal angle of resolution (logMAR) for the purpose of statistical analysis. Preoperative and postoperative visual acuities were compared using a paired Student’s t-test or a Wilcoxon signed rank test. The correlations of preoperative BCVA and MH size with postoperative BCVA were analyzed using Spearman’s correlation. A *P*-value < 0.05 was considered statistically significant.

## Results

The present study involved 10 eyes of 10 patients with full-thickness MHs (Table 1). This sample included two females and eight males, and the mean age was 23.30 ± 7.87 years (range: 13–39 years). Six patients (P1–P6) were injured by unexpected expose of handheld lasers when playing laser points by themselves or by others, and four patients (P7–P10) were injured by unexpected expose of YAG lasers when modulating the YAG laser transmitter in occupational settings. The mean interval from injury to surgery was 4.08 ± 3.6 months, and the mean follow-up time was 13.03 ± 18.06 months. The mean preoperative BCVA value was logMAR 0.86 ± 0.31 (range: 20/400 to 20/50). The mean preoperative MD and BD were 548.00 ± 286.10 μm and 1196.40 ± 524.41 μm, respectively.

**Table 1:** Clinical characteristics of patients with full-thickness MH caused by laser

Patient No.	Age	Sex	Laser type	Interval From Injury to Sugery	Preoperative BCVA	Tamponade agent used	MH Size (MD/BD) (μm)	Last follow-up After PPV (months)	BCVA at last follow-up	Post operative OCT
1*	39	M	laserpoint	2 mo	20/200	Air	606/1042	5 wk	20/100	Closed
2	16	M	laserpoint	6 mo	20/100	Air	358/982	11 mo	20/67	Closed;
3*	17	F	laserpoint	1 mo	20/167	Air	601/969	13 mo	20/50	Closed;
4*	25	F	laserpoint	2 mo	20/250	Air	966/2255	6 mo	20/200	Closed; destroyed RPE
5	23	M	Laserpoint	12 mo	20/50	C3F8	55/826	2 mo	20/25	Closed
6	13	M	laserpoint	4 mo	20/167	C3F8	857/2020	2 mo	20/40	Closed
7	25	M	Nd:YAG	3 wk	20/200	Air	823/1228	1 mo	20/100	Closed; destroyed RPE
8	22	M	Nd:YAG	3 mo	20/40	Air	294/602	3 mo	20/25	Closed;
9*	33	M	Nd:YAG	2 mo	20/200	C3F8	573/1041	54 mo	20/100	Closed; destroyed RPE
10	20	M	Nd:YAG	8 mo	20/400	SF6	347/999	37 mo	20/200	Closed; destroyed RPE

*Abbreviations*: *MH* macular hole, *M* Male, *F* female, *BCVA* Snellen best-corrected visual acuity, *C3F8* perfluoropropane, *SF6* sulfur hexafluoride, *MD* minimum diameter, *BD* base diameter, *PPV* pars plana vitrectomy, *OCT* optical coherence tomography;

*Inverted ILM flap technique was used

**Fig. 1:**
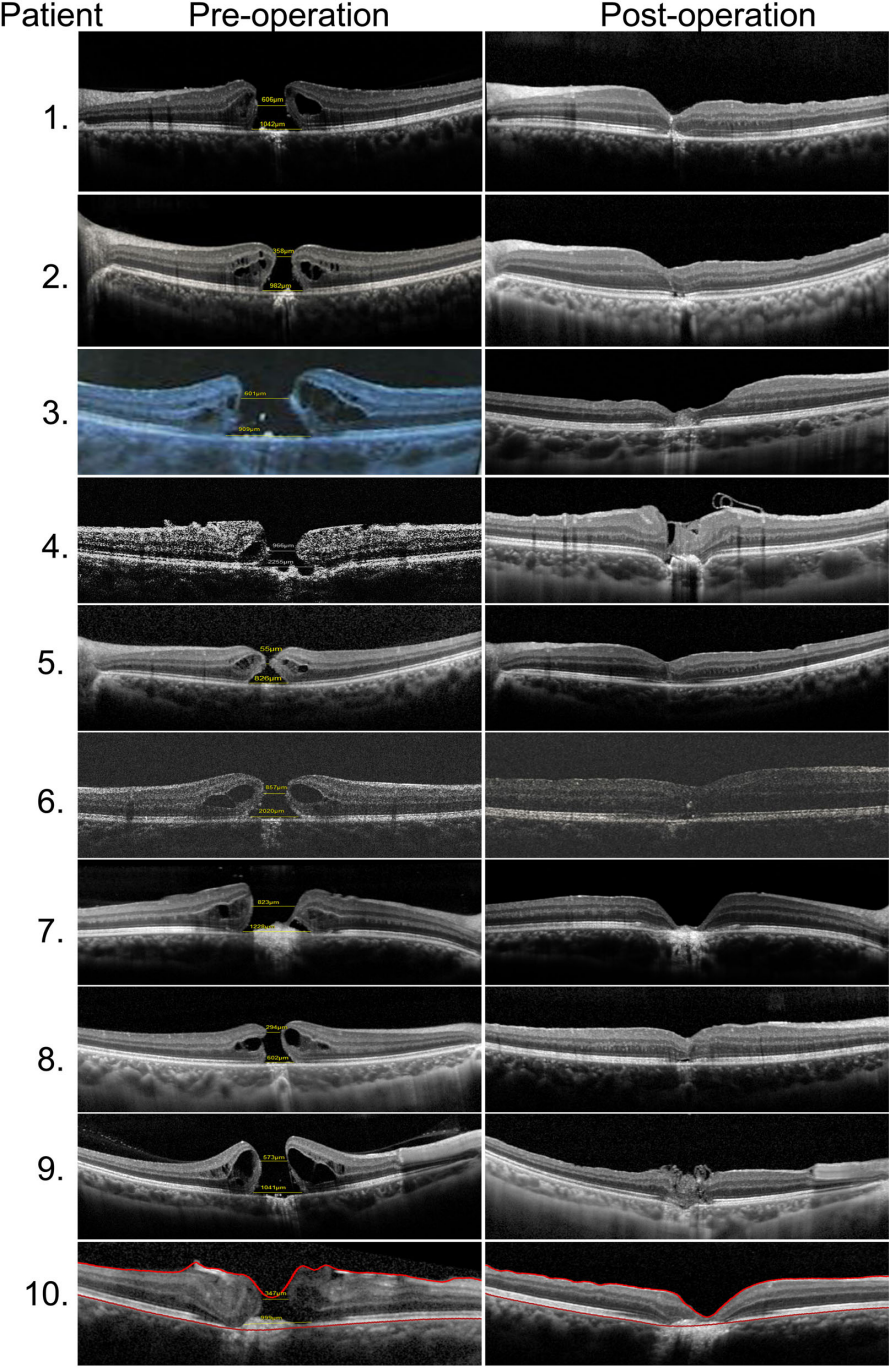
The macular structures of the 10 eyes before (left) and after (right) vitrectomy as shown by SD-OCT. Inverted ILM flap technique was used in P1, 3, 4 and 9. (Left) Ten patients exhibited full-thickness MHs preoperatively. Four patients (P4, P7, P9, and P10) exhibited obvious RPE disruption. (Right) In the three patients (P2, P5, and P8), ELM became continuous after surgery. At the last follow-up, all 10 eyes showed MH closure with various degrees of outer retina defects; four of these patients (P4, P7, P9, and P10) exhibited serious RPE destruction

**Fig. 2:**
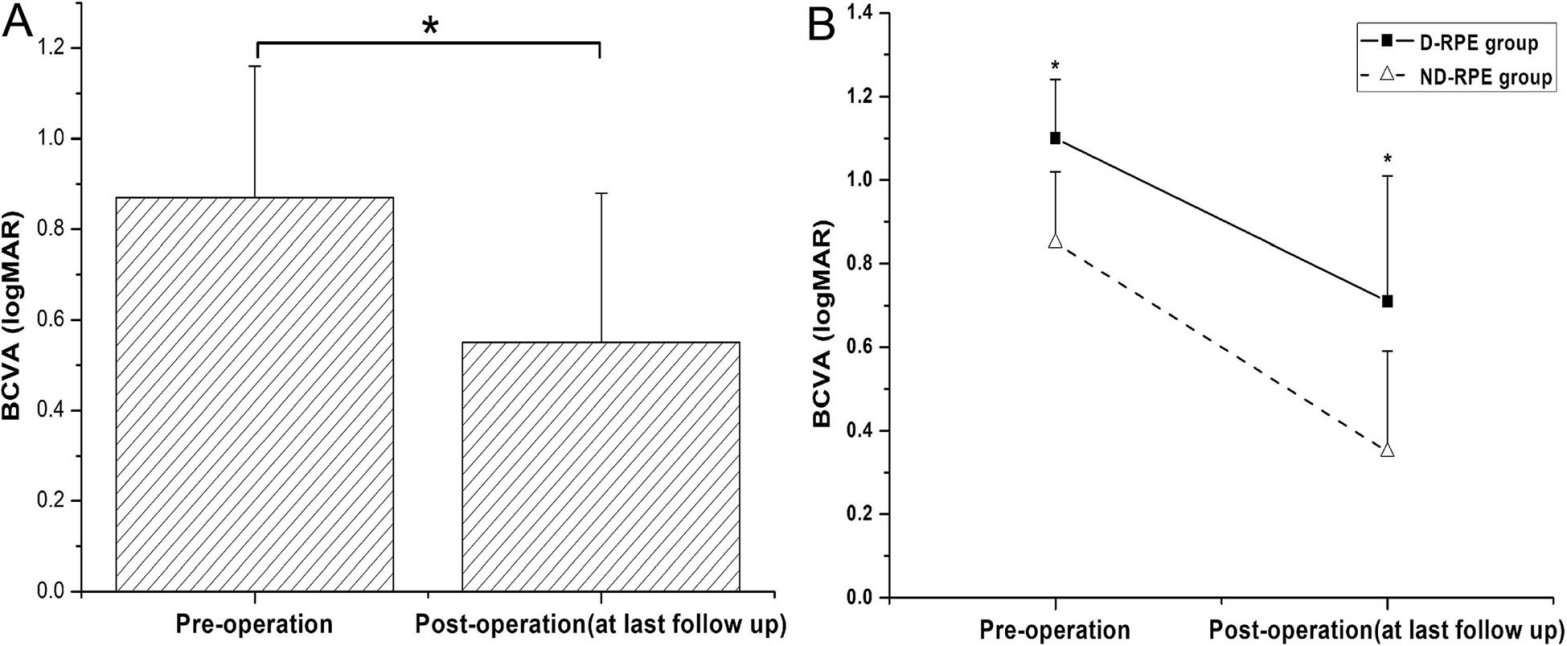
Changes of visual acuity before and after operation. The mean BCVA significantly improved after surgery at last follow-up in patients (*P* = 0.032; t-test) (**a**). The mean BCVA of D-RPE group was significantly worse than that of the ND-RPE group both before and after surgery (*P* = 0.019; Wilcoxon signed rank test). BCVA, Snellen best corrected visual acuity; D-RPE, destroyed RPE; ND-RPE, non-destroyed RPE. * *P* < 0.05 between the two groups

After 23-gauge PPV combined with ILM peeling, and gas tamponade (air, SF6, or C3F8), all 10 treated eyes (100%) showed MH closure at the last follow-up after operation (Fig. 1). The mean BCVA improved from logMAR 0.87 ± 0.29 (range: logMAR 0.3–1.3) to logMAR 0.55 ± 0.33 (range: logMAR 0.1–1.0; *P* = 0.032, t = 2.234) (Fig. 2a). Postoperative BCVA was significantly correlated with preoperative BCVA (*P* = 0.000, Spearman’s coefficient = 0.953) but was not correlated with MD or BD (*P* = 0.191, Spearman’s coefficient = 0.451; and *P* = 0.054, Spearman’s coefficient = 0.624, respectively). In P2, P5 and P8, external limiting membrane (ELM) became continuous after surgery. Postoperative BCVA was also significantly correlated with integrity of ELM after surgery (*P* = 0.039, Spearman’s coefficient = − 0.658).

Various degrees of outer retina defects were seen in all the eyes, of which four patients (P4, P7, P9, and P10) exhibited serious RPE destruction to the degree that the RPE layer could not be seen in SD-OCT. We divided all the patients into two groups: a destroyed RPE (D-RPE) group (P4, P7, P9, and P10) and a non-destroyed RPE (ND-RPE) group (P1, P2, P3, P5, P6, and P8). The mean preoperative BCVA values were logMAR 1.10 ± 0.14 and 0.71 ± 0.30 in the D-RPE and ND-RPE groups, respectively. The mean postoperative BCVA values were logMAR 0.85 ± 0.17 and 0.35 ± 0.24 in the D-RPE and ND-RPE groups, respectively. The mean BCVA of the former group was significantly worse than that of the latter group both before and after surgery (*P* = 0.019; Wilcoxon signed rank test) (Fig. 2b). Neither the mean BD or the MD between the D-RPE and ND-RPE groups had significant difference (*P* = 0.394; *P* = 0.163,respectively, Wilcoxon signed rank test). Furthermore, Both pre- and post-operative BCVA has a significant correlation with RPE destruction (*P* = 0.006, Spearman’s coefficient = 0.794).

**Fig. 3:**
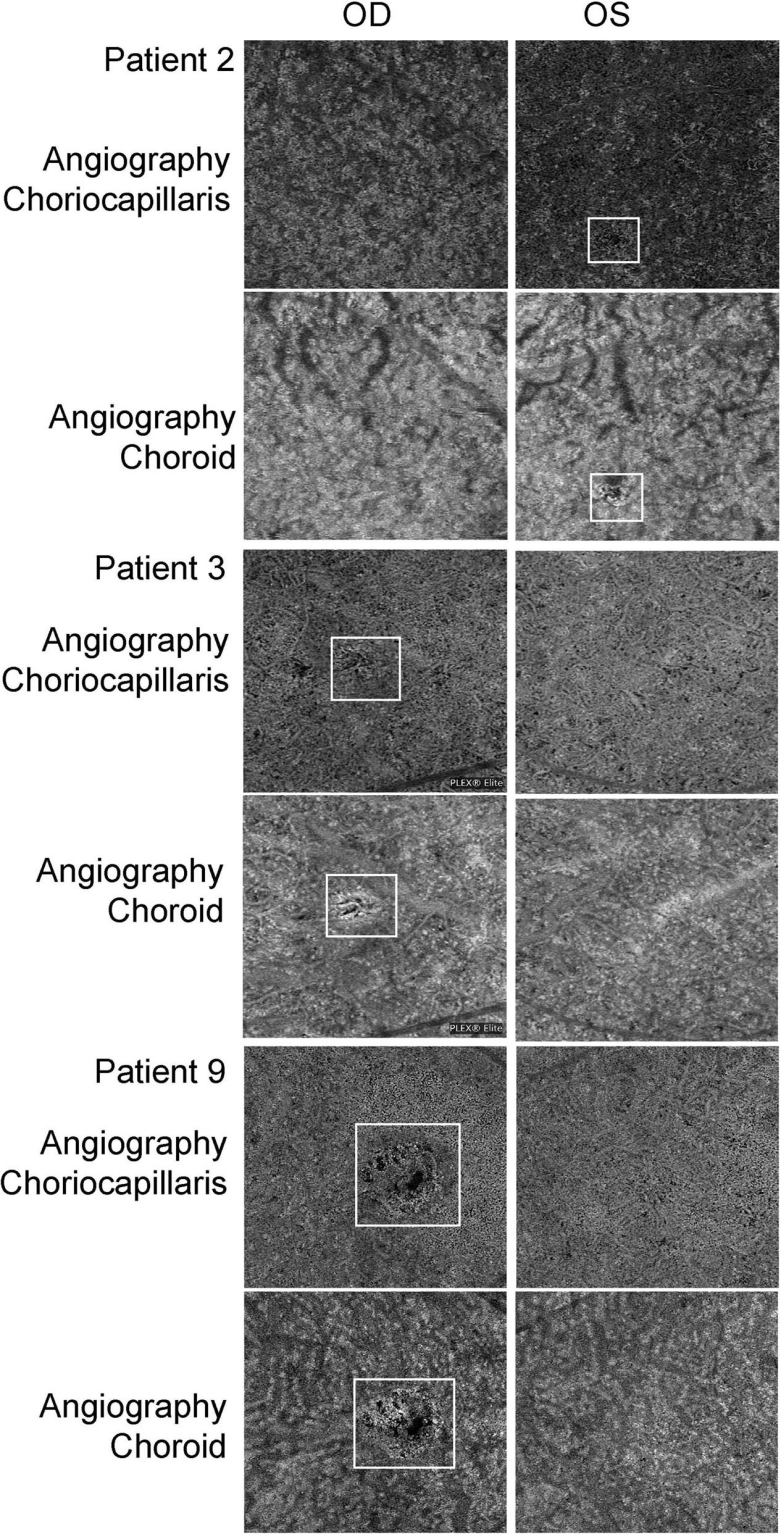
Swept-source OCTA images at the choriocapillaris and choroid levels of Patients 2, 3, and 9 at the last follow-up. The areas of flow deficit shown in the rectangular circle indicated choroidal ischemia in P2 (left eye), P3 (right eye), and P9 (right eye) in comparison to their fellow eyes

Through OCTA, we demonstrated choroidal ischemia caused by laser injury in Patients 2, 3, and 9.The areas of flow deficit demonstrated in the rectangular circle showed choroidal ischemia in P2 (left eye), P3 (right eye), and P9 (right eye) in comparison to their fellow eyes (Fig. 3), which indicated that the depth of laser damage to the eye was capable of reaching the choroid.

## Discussion

The results demonstrated that laser-induced MH responded well to vitrectomy,

with anatomic closure of the macular hole and improvement of visual acuity, though various degrees of outer retina defects remained. In addition, RPE and choroid damage might indicate poor visual outcomes.

Due to its thermal (e.g., laser pointer) and mechanical (e.g., Nd:YAG laser) effects on the retina, [9, 10] laser treatment can cause various forms of maculopathy, such as subretinal hemorrhage, outer retinal disruption, and full-thickness MHs [11–14].

Some studies have reported vitrectomy for laser-induced MHs [4–7, 15]; of these studies, only those by Alsulaiman and Qi have reported more than five cases of MHs. In Alsulaiman’s study, all MHs were caused by a handheld blue laser. And this study showed that 11 of the 14 treated eyes (78.6%) exhibited closure of the MH at the final follow-up; following closure of the MH, 8 of the 11 eyes showed different degrees of discontinuity of the outer layer of the retina [4]. In Qi’s study, MHs of five and six patients were caused by an Nd:YAG laser and handheld lasers respectively. All 11 operated eyes (100%) in Qi’s study showed MH closure at the last follow-up after operation, but variable degrees of loss of outer layer of the retina remained, and even part of choroidal defect occurred in four patients in OCT [5].

All 10 eyes (100%) in the present study showed closure of the MH with variable degrees disruption of outer layer of retina after surgery. The mean MD of MH in the present case series was 548.00 ± 286.10 μm, larger than those of Alsulaiman’s (351 ± 151.7 μm) and Qi’s (505.5 ± 163.0 μm) studies. It was worth noting that in four eyes of our cases, the RPE layers were completely unrecognized in OCT, indicating more serious laser damage. Unlike idiopathic MHs, laser-induced MHs could be accompanied by RPE damage and choroidal ischemia. Similarly, RPE disruption has also been reported to accompany MHs in previous studies [16]. Further, OCTA of the three eyes in our study demonstrated that choroidal ischemia caused by laser injury, indicating the depth of laser damage to the eye, was capable of reaching the choroid level. As far as we knew, there were few reports on OCTA of laser-induced MHs except ours. Similarly, such inner choroidal ischemia demonstrated by OCTA was reported in a case of choroidal neovascularization, but not MH, due to handheld laser-induced maculopathy [17]. OCTA could provide more information about the mechanism of laser-induced MH, and hence, perhaps, it should be used as a routine examination to help assess the severity of such a special subtype of MH.

Good preoperative visual acuity and no severe RPE and choroid damage might suggest better postoperative visual acuity. Postoperative BCVA was improved in all patients and was only significantly correlated with the preoperative BCVA, not with preoperative MD or BD of the MH. Similarly, in a study by Qi, postoperative BCVA was not correlated with the preoperative size of the MH [5]. Further, the mean BCVA of the D-RPE group was significantly worse than that of the ND-RPE group both preoperatively and postoperatively. And both pre- and post-operative BCVA was significantly correlated with RPE destruction. Thus, we inferred that RPE and choroidal damage may play an important role in the pathophysiological mechanism of laser-induced MH and may also account for the unsatisfactory recovery of macular structure and BCVA after vitrectomy.

There are some limitations in this study. For example, it’s a retrospective study and the number of cases is small. However, this study enriches the understanding of surgical outcomes of macular hole caused by laser injury.

In summary, this study supported that laser-induced macular hole could be treated by vitrectomy combined with ILM peeling and gas tamponade. Postoperative BCVA may be correlated with preoperative BCVA. Finally, If RPE/choroid is involved in laser damage in addition to the outer retinal layer, this may indicate poor visual prognosis.

## Data Availability

The datasets used and/or analysed during the current study available from the corresponding author on reasonable request.

## References

[1] Petrou P, Patwary S, Banerjee PJ, Kirkby GR (2014). Bilateral macular hole from a handheld laser pointer. Lancet.

[2] Androudi S, Papageorgiou E. Macular hole from a laser pointer. N Engl J Med. 2018;378(25):2420–0. 10.1056/NEJMicm1714488 29924946

[3] Shuai Y, Chen X, Fang W, Li J, Ge W, Yuan S (2017). Focal choroidal excavation and a traumatic macular hole secondary to accidental Q-switched Nd:YAG laser. Photodiagn Photodyn Ther.

[4] Alsulaiman SM, Alrushood AA, Almasaud J, Alkharashi AS, Alzahrani Y, Abboud EB (2015). Full-thickness macular hole secondary to High-power handheld blue laser: natural history and Management outcomes. Am J Ophthalmol.

[5] Qi Y, Wang Y, You Q, Tsai F, Liu W (2017). Surgical treatment and optical coherence tomographic evaluation for accidental laser-induced full-thickness macular holes. Eye (Lond).

[6] Botsford BW, Williams AM, Martel JN (2020). High-powered blue-light laser-induced MACULOPATHY in an adolescent: multi-modal imaging, evolution, and MANAGEMENT. Retinal Cases Brief Rep.

[7] Yang YH, Chung YT, Kim BK, Moon JH, Mun SJ (2019). Inverted internal limiting membrane flap technique and an autologous platelet concentrate to treat an Nd:YAG laser-induced macular hole. A case report. Medicine.

[8] Michalewska Z, Michalewski J, Adelman RA, Nawrocki J (2010). Inverted internal limiting membrane flap technique for large macular holes. Ophthalmology.

[9] Barkana Y, Belkin M (2000). Laser eye injuries. Surv Ophthalmol.

[10] Mainster MA (2004). Assessment of alleged retinal laser injuries. Arch Ophthalmol.

[11] Alsulaiman SM, Alrushood AA, Almasaud J, Alzaaidi S, Alzahrani Y, Arevalo JF (2014). High-power handheld blue laser-induced Maculopathy. Ophthalmology.

[12] Raoof N, Bradley P, Theodorou M, Moore AT, Michaelides M (2016). The new pretender: a large UK case series of retinal injuries in children secondary to handheld lasers. Am J Ophthalmol.

[13] Wyrsch S, Baenninger PB, Schmid MK (2010). Retinal injuries from a handheld laser pointer. N Engl J Med.

[14] Raoof N, Chan TK, Rogers NK, Abdullah W, Haq I, Kelly SP (2014). 'Toy' laser macular burns in children. Eye (Lond).

[15] Sou R, Kusaka S, Ohji M, Gomi F, Ikuno Y, Tano Y (2003). Optical coherence tomographic evaluation of a surgically treated traumatic macular hole secondary to Nd:YAG laser injury. Am J Ophthalmol.

[16] Lee GD, Baumal CR, Lally D, Pitcher JD, Vander J, Duker JS (2014). Retinal injury after inadvertent handheld laser exposure. Retina.

[17] Tran K, Wang D, Scharf J, Sadda S, Sarraf D (2020). Inner choroidal ischaemia and CNV due to handheld laser-induced maculopathy: a case report and review. Eye.

